# Global burden of gout among young people from 1990 to 2021, with projections for 2050: A systematic analysis based on the Global Burden of Disease Study 2021

**DOI:** 10.1371/journal.pone.0333368

**Published:** 2025-10-29

**Authors:** Peipei Tian, Hailiang Zhao, Botao Wang, Yanpeng Chen, Zhijun Jia, Chao Wang, Guangya Wang

**Affiliations:** 1 Department of Endocrinology and Metabolism, Cangzhou Central Hospital, Cangzhou, Hebei, China; 2 Quality Management and Control Office, The Hebei Medical University Third Hospital, Shijiazhuang, Hebei, China; 3 Medical Administration Department, Cangzhou Central Hospital, Cangzhou, Hebei, China; 4 Radiology Intervention Department, Cangzhou Central Hospital, Cangzhou, Hebei, China; 5 Chinese Medicine Hall, Cangzhou Central Hospital, Cangzhou, Hebei, China; 6 Hebei Key Laboratory of Metabolic Diseases, Hebei General Hospital, Shijiazhuang, Hebei, China; Prime Hospital LLC, UNITED ARAB EMIRATES

## Abstract

Gout represents the most common form of inflammatory arthritis, exerting a substantial impact on patient quality of life and productivity. In recent years, the age distribution of gout has shifted, with an increasing incidence among younger individuals. Accurate statistics regarding the incidence, prevalence, and disability-adjusted life years (DALYs) associated with gout in this population are critical for effective resource allocation in prevention and treatment strategies. In the current study, data from the 2021 Global Burden of Disease (GBD) database were used to analyze the burden of gout among young people aged 15–44 years in 204 countries and territories from 1990 to 2021, with projections extending over the next 25 years. Data on prevalence, incidence, and DALYs were collected and stratified by geographical location, gender, and economic development level. Decomposition analysis and predictive modeling were employed to evaluate trends and the impact of critical indicators. In 2021, 8,516,229 young individuals were affected by gout globally, with 1,975,165 new cases and DALYs totaling 283,725 person-years. Since 1990, the age-standardized prevalence rate, age-standardized incidence rate, and age-standardized DALYs rate increased by 10.4%, 9.4%, and 10.2%, respectively, all showing positive correlation with the sociodemographic index (*r* = 0.319, 0.272, 0.320, *P* < 0.001). It is projected that by 2046, global figures in young people will reach approximately 9,229,870 total cases, 2,202,802 new cases, and 307,441 DALYs. Males are anticipated to account for 7,460,349 affected individuals, 1,762,412 new cases, and 245,542 DALYs, whereas females will represent 1,769,521 affected individuals, 440,390 new cases, and 61,899 DALYs. The global disease burden of gout among young people increased significantly from 1990 to 2021, and it is expected to continue rising over the next 25 years. Comprehensive epidemiological data remain essential for guiding gout prevention and management, as well as shaping effective health policies.

## Introduction

Gout, a form of crystalline arthropathy, results from impaired uric acid excretion, causing monosodium urate deposition within joints. Clinical manifestations include redness, swelling, heat, and pain. It represents the most common form of inflammatory arthritis [1]. Current global estimates of gout prevalence range from 1% to 4% [2], with approximately 55.8 million people worldwide affected by gout in 2020 [3].

Prospective studies have demonstrated that men diagnosed with gout exhibit increased mortality risks across various causes. Notably, among men without a history of coronary heart disease (CHD), this elevated risk is primarily attributed to a greater susceptibility to cardiovascular disease, particularly CHD [4]. A persistent mortality gap exists in all-cause and cardiovascular mortality among individuals with gout, reinforcing the role of gout-specific pathways, such as flare-induced inflammation, in these outcomes [5]. Furthermore, gout is independently associated with increased mortality due to renal disease [6] and a transiently increased risk of cardiovascular events following acute attacks [7].

With rapid economic development and improved living standards, gout is being diagnosed more frequently in younger populations. Gout demonstrates notable sex disparity in younger populations, being exceptionally rare in premenopausal women due to estrogen’s ability to enhance renal urate excretion [8,9]. This epidemiological pattern highlights the importance of researching early-onset gout to identify modifiable risk factors beyond hormonal influences. Consequently, gout is emerging as a major public health concern worldwide, impacting quality of life and imposing a significant burden on global healthcare systems [10,11]. Understanding the disease burden of gout among young people worldwide is crucial for ensuring the allocation of adequate medical resources across regions. While earlier studies on the global burden of gout primarily relied on data collected before 2019, more recent analyses, including the Global Burden of Disease (GBD) Study 2021 (GBD 2021), offer updated estimates through 2021 [3].

Gout not only adversely impacts patients’ quality of life and work productivity but also imposes a substantial economic burden, thereby straining global healthcare systems. With rapid economic development, shifts in lifestyle habits and dietary patterns have contributed to rising gout incidence rates, while the trend toward younger onset age warrants urgent attention. Therefore, this study utilizes data from the Institute for Health Metrics and Evaluation GBD database to analyze trends in incidence, mortality, and disability-adjusted life years (DALYs) associated with gout in young people from 1990 to 2021, as well as to predict potential trends in disease burden over the next 25 years.

## Methods

### Data source

The data utilized in this study were obtained from the Global Health Data Exchange GBD Results Tool (http://ghdx.healthdata.org/gbd-results-tool). Physician-diagnosed gout based on the criteria of the American College of Rheumatology (ACR) was used as the reference case definition in GBD [12]. Data sources that used diagnostic criteria other than the reference criteria, such as self-reported or administrative gout data, were adjusted with the MR-BRT (Meta-Regression Bayesian Regularized Trimmed) meta-regression tool [13]. Data on the prevalence, incidence, and DALYs of gout among young individuals aged 15–44 years from 1990 to 2021 were collected for 204 countries and territories. This age group represents the working-age population most vulnerable to gout’s impact on productivity, while excluding older adults with potentially confounding comorbidities. Analyses considered geographical location, socioeconomic development, and sex. Results are presented as numbers, rates, and percentages, with all rates calculated per 100,000 population. As the GBD database is public, ethical approval was not required.

The prevalence, incidence, and DALYs used in this study serve as fundamental indicators for measuring the epidemiological trends of diseases. Accurate assessment of the global burden of gout necessitates metrics that account for variations in population age structure, particularly when conducting comparisons between countries at different development stages. Age-standardized rates (ASRs) are regarded as the gold standard for such analyses, as they control for confounding effects arising from demographic differences [GBD 2021 IHME (2025). VizHub-GBD results. Available online at: https://vizhub.healthdata.org/gbd-results/.]. The age-standardized prevalence rate (ASPR) can offer a precise measurement of disease (gout) burden across diverse populations, while the age-standardized incidence rate (ASIR) captures trends related to early-onset gout. The age-standardized DALYs rate (ASDR) can provide quantitative insights into mortality and morbidity associated with gout. Collectively, these metrics facilitated the robust quantification of gout’s impact on young populations globally.

The Socio-Demographic Index (SDI) is a standardized metric used to evaluate the developmental status of countries or regions based on fertility rates, education levels, and per capita income. The SDI is scored on a scale from 0 to 1, with higher values reflecting greater socioeconomic development. The SDI is associated with disease incidence and mortality rates.

### Statistical analysis

The temporal trends for gout among young individuals were analyzed from 1990 to 2021 using average annual percentage changes (AAPC) calculated by Joinpoint 5.2.0.0 (Statistical Research and Applications Branch, National Cancer Institute, USA).

ASRs were derived by stratifying gout cases and DALYs into 5-year age groups (15–19, 20–24,..., 40–44 years), adjusting the rates to the GBD 2021 standard population, and using direct standardization to minimize bias from age distribution across countries (Eq. (1)):


$$ASR=\;\frac{\sum \left(a_{i}\times w_{i}\right)}{\sum w_{i}}\times \text{1}\text{0}\text{0},\text{0}\text{0}\text{0}$$ (1)

where *a*_*i*_ is the age-specific rate, and *w*_*i*_ *is* the standard population weight for age group *i*.

Future estimates for prevalence, incidence, and DALYs—as counts and rates—in patients with gout aged 15–44 were forecasted for the next 25 years using the Bayesian Age-Period-Cohort (BAPC) model (version 0.0.36) in R (version 4.4.3), stratified by sex and total population. The BAPC model was selected due to several features described below:

Disentangling temporal drivers: The BAPC model offers a robust framework for separating the effects of age (15–44 years), time period, and birth cohort. Trends in gout epidemiology and burden within this working-age population are likely influenced by factors acting through these dimensions (e.g., aging within the bracket, evolving diagnostic practices/awareness over time, generational differences in risk factors).

Stratified forecasting: The model supports stratified forecasts, facilitating distinct, coherent projections for males, females, and the total group.

Comprehensive output: The model enables simultaneous forecasting of absolute burden metrics (prevalence, incidence, DALY counts) and population-adjusted rates (prevalence, incidence, DALY rates), providing a complete overview of future gout trends.

Bayesian inference was conducted using integrated nested Laplace approximation (INLA) due to its computational efficiency in approximating posterior distributions, which is suitable for modeling complex hierarchical structures and large datasets. Forecasts were derived from the projected posterior distributions, yielding age-specific estimates (ages 15–44) of prevalence, incidence, and DALYs, as both counts and rates, stratified by sex (male, female), as well as the total population.

The relationship between global gout burden and SDI among young people in 2021 was assessed using the Spearman rank test, while Das Gupta’s decomposition method was applied to quantify the relative impact of age structure, population growth, and epidemiological changes [14]. Predictive analyses covered 1990–2021 and extended 25 years ahead to provide insights for improving future gout-related DALY rates. Finally, a descriptive and visual analysis of the global gout burden among young individuals from 1990 to 2021 was conducted, categorizing the results by sex, region, and year, with statistical significance set at *P *< 0.05.

## Results

### Global burden of gout in young people across 204 countries and territories (1990–2021): Prevalence, incidence, DALYs, and Temporal Trends

In 1990, 4,813,482.01 young people had gout across 204 countries and territories, with a crude prevalence rate of 194.23 per 100,000 persons and an ASPR of 217.70 (95% confidence interval [CI]: 217.51–217.90) per 100,000 persons. The number of incident cases was 1,150,836.54, with a crude incidence rate of 46.44 per 100,000 persons per year and an ASIR of 51.05 (95% CI: 50.95–51.14) per 100,000 persons. The number of DALYs was 160,884.26 person-years, with a crude DALY rate of 6.49 per 100,000 person-years and an ASDR of 7.27 (95% CI: 7.23–7.30) per 100,000 person-years.

**Fig 1:**
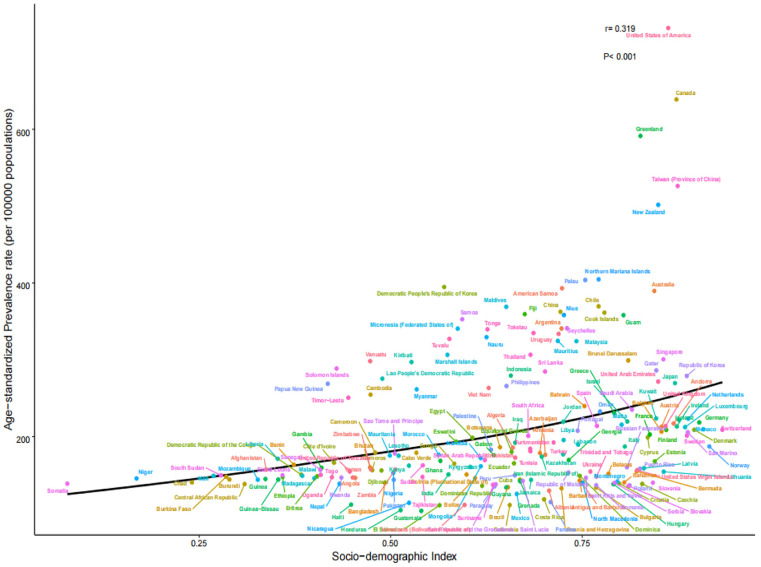
ASPR of 204 countries and territories in 2021. ASPR, age-standardized prevalence rate.

**Fig 2:**
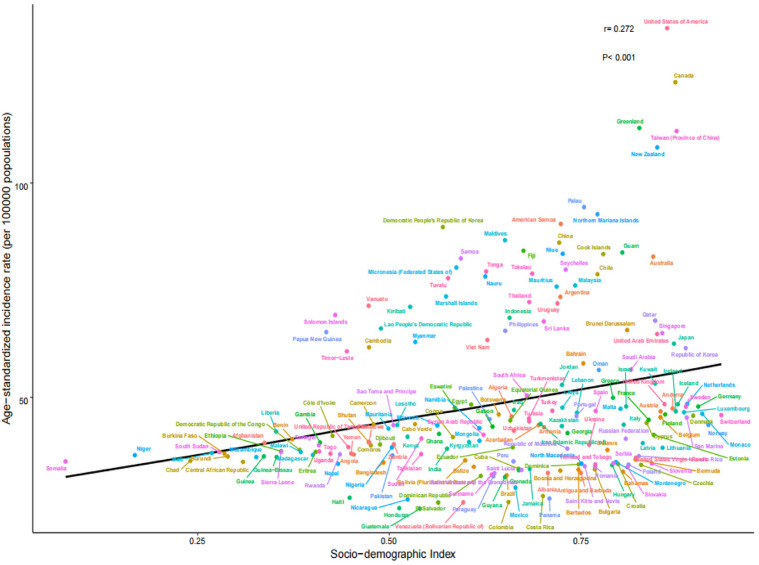
ASIR of 204 countries and territories in 2021. ASIR, age-standardized incidence rate.

**Fig 3:**
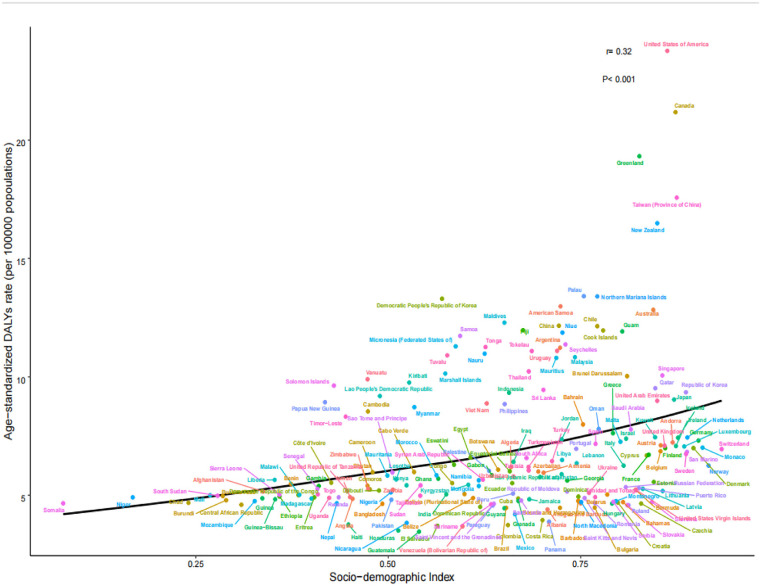
ASDR of 204 countries and territories in 2021. ASDR, age-standardized DALYs rate.

By 2021, the number of young people with gout across 204 countries and territories had increased to 8,516,229.01, with a crude prevalence rate of 245.07 per 100,000 persons and an ASPR of 240.41 (95% CI: 240.25–240.57) per 100,000 persons, representing a 10.4% since 1990, with an AAPC of 0.33 (95% CI: 0.32–0.33; Table 1). The number of incident cases rose to 1,975,165.71, with a crude incidence rate of 56.84 per 100,000 persons and an ASIR of 55.86 (95% CI: 55.78–55.94) per 100,000 persons, representing a 9.4% increase since 1990, with an AAPC of 0.29 (95% CI: 0.29–0.30; Table 2). The number of DALYs increased to 283,725.7, with a crude DALY rate of 8.16 per 100,000 person-years and an ASDR of 8.01 (95% CI: 7.98–8.04) per 100,000 person-years, representing a 10.2% increase since 1990, with an AAPC of 0.32 (95% CI: 0.31–0.33; Table 3). ASPR, ASIR, and ASDR positively correlated with the SDI (*r* = 0.319, 0.272, and 0.320, respectively; *P* < 0.001). From 1990 to 2021, the gout-related disease burden steadily increased (Figs 1–3).

**Table 1:** Prevalence number and ASPR of gout in 1990 and 2021 and temporal trends from 1990 to 2021.

Characteristic	1990			2021			1990–2021
	Prevalence	Crude rate per 100,000	ASPR per 100,000 NO. (95% CI)	Prevalence number	Crude rate per 100,000	ASPR per 100,000 NO. (95% CI)	AAPC NO. (95% CI)
Global	4,813,482.01	194.23	217.70 (217.51–217.90)	8,516,229.01	245.07	240.41 (240.25–240.57)	0.33 (0.32–0.33)
Female	934,933.00	76.41	85.37 (85.19–85.54)	1,601,331.27	93.47	91.36 (91.22–91.51)	0.23 (0.23–0.24)
Male	3,878,549.02	309.12	345.79 (345.45–346.14)	6,914,897.76	392.48	386.67 (386.38–386.96)	0.36 (0.36–0.37)
Socio-demographic Index							
Low SDI	226,063.12	110.69	138.58 (138.01–139.16)	596,653.93	119.17	147.02 (146.64–147.39)	0.19 (0.19–0.20)
Low-middle SDI	625,490.09	123.50	148.62 (148.25–148.99)	1,400,866.05	152.64	164.10 (163.83–164.37)	0.33 (0.32–0.33)
Middle SDI	1,568,171.63	186.64	221.98 (221.63–222.33)	2,7415,17.50	250.96	236.54 (236.26–236.82)	0.21 (0.20–0.22)
High-middle SDI	1,137,276.51	220.83	233.50 (233.07–233.93)	1,758,039.07	330.04	280.73 (280.31–281.15)	0.60 (0.58–0.61)
High SDI	1,253,354.73	305.61	288.37 (287.86–288.87)	2,013,948.75	469.60	406.07 (405.51–406.63)	1.11 (1.10–1.12)
Region							
Andean Latin America	16,645.63	96.65	116.00 (114.22–117.79)	47,199.23	150.75	153.84 (152.45–155.23)	0.90 (0.89–0.91)
Australasia	30,940.36	321.06	307.25 (303.84–310.70)	56,168.67	450.81	407.64 (404.27–411.03)	0.91 (0.91–0.92)
Caribbean	15,565.49	93.14	107.46 (105.76–109.17)	28,018.38	132.86	133.23 (131.67–134.80)	0.70 (0.70–0.71)
Central Asia	37,681.26	121.35	147.53 (145.99–149.08)	77,208.47	177.84	171.20 (169.99–172.42)	0.48 (0.48–0.48)
Central Europe	70,147.89	126.72	118.59 (117.71–119.47)	74,658.04	169.61	136.80 (135.81–137.80)	0.45 (0.45–0.46)
Central Latin America	60,517.78	79.91	95.91 (95.14–96.69)	138,407.81	117.33	117.98 (117.36–118.61)	0.67 (0.67–0.68)
Central Sub-Saharan Africa	24,744.64	108.94	142.06 (140.26–143.89)	73,321.90	121.76	150.32 (149.22–151.42)	0.18 (0.18–0.19)
East Asia	1,720,710.52	270.92	308.64 (308.18–309.11)	2,465,917.95	429.27	365.89 (365.43–366.35)	0.55 (0.53–0.56)
Eastern Europe	14,9851.90	150.49	142.31 (141.59–143.03)	162,858.98	199.23	158.35 (157.56–159.13)	0.34 (0.33–0.34)
Eastern Sub-Saharan Africa	81,795.99	105.25	139.58 (138.61–140.56)	230,725.47	118.65	149.97 (149.35–150.59)	0.24 (0.23–0.24)
High-income Asia Pacific	212,055.51	261.07	245.59 (244.55–246.65)	214,255.62	336.80	274.10 (272.92–275.28)	0.35 (0.35–0.36)
High-income North America	61,5245.76	461.09	430.03 (428.96–431.11)	1,141,753.91	778.64	722.26 (720.93–723.58)	1.68 (1.65–1.70)
North Africa and Middle East	191,123.24	128.74	160.22 (159.49–160.94)	594,283.04	199.34	192.07 (191.58–192.56)	0.59 (0.58–0.59)
Oceania	6,498.77	219.92	268.93 (262.36–275.63)	16,527.76	257.57	282.40 (278.10–286.75)	0.16 (0.15–0.17)
South Asia	577,054.44	119.02	139.36 (139.00–139.72)	1,274,572.11	140.52	148.74 (148.49–149.00)	0.21 (0.21–0.22)
Southeast Asia	418,354.47	191.37	230.64 (229.93–231.35)	936,444.85	286.91	277.88 (277.31–278.44)	0.61 (0.60–0.61)
Southern Latin America	58,300.74	265.32	280.02 (277.75–282.30)	110,587.24	363.54	348.25 (346.20–350.31)	0.70 (0.70–0.71)
Southern Sub-Saharan Africa	33,022.38	138.08	173.72 (171.83–175.63)	73,739.86	188.72	192.31 (190.92–193.71)	0.33 (0.32–0.33)
Tropical Latin America	67,026.38	92.79	105.60 (104.8–106.41)	152,328.54	144.72	134.09 (133.41–134.76)	0.77 (0.77–0.78)
Western Europe	333,793.67	195.64	187.29 (186.65–187.93)	384,401.09	242.15	209.14 (208.48–209.81)	0.36 (0.35–0.37)
Western Sub-Saharan Africa	92,405.19	116.70	148.33 (147.36–149.30)	262,850.10	123.84	156.30 (155.70–156.91)	0.19 (0.18–0.20)

ASPR, age-standardized prevalence rate; CI, confidence interval; AAPC, average annual percentage change.

**Table 2:** Incidence number and ASIR of gout in 1990 and 2021 and temporal trends from 1990 to 2021.

Characteristic	1990			2021			1990–2021
	Incidence	Crude rate per 100,000	ASIR per 100,000 NO. (95% CI)	Incidence	Crude rate per 100,000	ASIR per 100,000 NO. (95% CI)	AAPC NO. (95% CI)
Global	1,150,836.54	46.44	51.05 (50.95–51.14)	1,975,165.71	56.84	55.86 (55.78–55.94)	0.29 (0.29–0.30)
Female	229,626.27	18.77	20.59 (20.5–20.67)	389,707.38	22.75	22.29 (22.22–22.36)	0.27 (0.26–0.27)
Male	921,210.27	73.42	80.58 (80.42–80.75)	1,585,458.33	89.99	88.77 (88.63–88.91)	0.32 (0.31–0.32)
Socio-demographic Index							
Low SDI	58,442.70	28.62	34.77 (34.49–35.06)	154,056.18	30.77	36.96 (36.78–37.15)	0.20 (0.20–0.21)
Low-middle SDI	159,343.55	31.46	36.89 (36.71–37.08)	350,925.42	38.24	40.67 (40.54–40.81)	0.32 (0.31–0.32)
Middle SDI	389,130.57	46.31	53.52 (53.35–53.69)	655,869.84	60.04	56.97 (56.83–57.10)	0.21 (0.20–0.22)
High-middle SDI	275,773.59	53.55	55.89 (55.68–56.10)	409,149.14	76.81	66.68 (66.47–66.89)	0.57 (0.55–0.58)
High SDI	267,379.15	65.20	61.93 (61.69–62.16)	403,916.27	94.18	83.55 (83.29–83.81)	0.96 (0.93–0.99)
Region							
Andean Latin America	4,045.29	23.49	27.20 (26.36–28.07)	11,125.03	35.53	35.98 (35.32–36.66)	0.90 (0.88–0.92)
Australasia	6,769.31	70.24	67.67 (66.07–69.31)	11,842.21	95.05	86.99 (85.43–88.58)	0.80 (0.78–0.81)
Caribbean	3,744.28	22.40	25.05 (24.25–25.88)	6,565.84	31.13	31.12 (30.37–31.89)	0.70 (0.70–0.71)
Central Asia	9,603.36	30.93	36.20 (35.45–36.96)	18,933.42	43.61	41.97 (41.37–42.57)	0.48 (0.47–0.48)
Central Europe	17,284.32	31.22	29.52 (29.08–29.96)	18,101.51	41.12	34.12 (33.61–34.63)	0.46 (0.45–0.47)
Central Latin America	14,446.18	19.08	21.97 (21.60–22.33)	31,994.07	27.12	27.23 (26.94–27.53)	0.70 (0.69–0.71)
Central Sub-Saharan Africa	6,430.77	28.31	35.49 (34.61–36.4)	18,902.68	31.39	37.70 (37.16–38.25)	0.20 (0.19–0.20)
East Asia	419,564.74	66.06	73.49 (73.27–73.72)	572,720.96	99.70	86.61 (86.38–86.83)	0.54 (0.53–0.55)
Eastern Europe	37,528.14	37.69	35.73 (35.37–36.10)	39,937.47	48.86	39.89 (39.49–40.29)	0.35 (0.34–0.36)
Eastern Sub-Saharan Africa	21,359.28	27.48	35.09 (34.61–35.57)	59,758.34	30.73	37.70 (37.39–38.01)	0.24 (0.23–0.24)
High-income Asia Pacific	47,971.89	59.06	56.41 (55.91–56.92)	47,128.53	74.08	62.34 (61.77–62.92)	0.32 (0.31–0.33)
High-income North America	121,545.04	91.09	85.48 (84.99–85.96)	209,850.31	143.11	134.81 (134.23–135.38)	1.47 (1.41–1.52)
North Africa and Middle East	48,531.46	32.69	39.45 (39.09–39.80)	144,797.36	48.57	47.04 (46.80–47.28)	0.57 (0.56–0.58)
Oceania	1,620.28	54.83	64.84 (61.68–68.13)	4,045.81	63.05	68.02 (65.93–70.16)	0.16 (0.15–0.17)
South Asia	148,324.49	30.59	35.07 (34.89–35.25)	324,285.67	35.75	37.53 (37.40–37.66)	0.22 (0.22–0.23)
Southeast Asia	105,433.46	48.23	56.29 (55.95–56.64)	226,160.58	69.29	67.37 (67.10–67.65)	0.58 (0.58–0.59)
Southern Latin America	12,776.23	58.14	60.85 (59.80–61.92)	23,652.77	77.76	74.84 (73.88–75.80)	0.65 (0.63–0.67)
Southern Sub-Saharan Africa	8,540.39	35.71	43.54 (42.61–44.49)	18,565.92	47.51	48.18 (47.49–48.88)	0.32 (0.32–0.33)
Tropical Latin America	16,255.86	22.50	24.93 (24.55–25.32)	35,696.42	33.91	31.82 (31.49–32.15)	0.79 (0.78–0.79)
Western Europe	75,304.97	44.14	42.44 (42.14–42.75)	83,624.01	52.68	46.58 (46.26–46.90)	0.28 (0.27–0.29)
Western Sub-Saharan Africa	23,756.84	30.00	36.93 (36.46–37.41)	67,476.80	31.79	38.93 (38.63–39.23)	0.18 (0.17–0.19)

ASIR, age-standardized incidence rate; CI, confidence interval; AAPC, average annual percentage change.

**Table 3:** DALY number and ASDR of gout in 1990 and 2021 and temporal trends from 1990 to 2021.

Characteristic	1990			2021			1990–2021
	DALYs	Crude rate per 100,000	ASDR per 100,000 NO. (95% CI)	DALYs	Crude rate per 100,000	ASDR per 100,000 NO. (95% CI)	AAPC NO. (95% CI)
Global	160,884.26	6.49	7.27 (7.23–7.3)	283,725.7	8.16	8.01 (7.98–8.04)	0.32 (0.31–0.33)
Female	31,460.61	2.57	2.87 (2.83–2.9)	53,660.44	3.13	3.06 (3.04–3.09)	0.23 (0.22–0.24)
Male	129,423.66	10.31	11.53 (11.46–11.59)	230,065.28	13.06	12.86 (12.81–12.92)	0.36 (0.35–0.37)
Socio-demographic Index							
Low SDI	7,544.73	3.69	4.61 (4.51–4.72)	20,000.65	3.99	4.92 (4.85–4.99)	0.21 (0.2–0.22)
Low-middle SDI	21,007.56	4.15	4.98 (4.91–5.05)	46,880.42	5.11	5.49 (5.44–5.54)	0.32 (0.31–0.33)
Middle SDI	52,574.35	6.26	7.43 (7.36–7.49)	91,688.89	8.39	7.91 (7.86–7.96)	0.21 (0.19–0.22)
High-middle SDI	38,109.05	7.4	7.81 (7.74–7.89)	5,8776	11.03	9.40 (9.32–9.47)	0.6 (0.58–0.62)
High SDI	41,543.68	10.13	9.56 (9.47–9.65)	66,205.67	15.44	13.36 (13.26–13.47)	1.09 (1.08–1.1)
Region							
Andean Latin America	562.58	3.27	3.91 (3.59–4.25)	1,590	5.08	5.18 (4.93–5.44)	0.89 (0.85–0.92)
Australasia	1,023.35	10.62	10.16 (9.55–10.81)	1,848.17	14.83	13.42 (12.82–14.05)	0.88 (0.85–0.91)
Caribbean	526.5	3.15	3.63 (3.32–3.95)	940.42	4.46	4.47 (4.19–4.77)	0.68 (0.67–0.69)
Central Asia	1,268.77	4.09	4.95 (4.67–5.25)	2,590.63	5.97	5.74 (5.52–5.97)	0.48 (0.47–0.5)
Central Europe	2,358.24	4.26	3.99 (3.83–4.16)	2,512.68	5.71	4.61 (4.43–4.8)	0.46 (0.45–0.47)
Central Latin America	2,043.32	2.7	3.23 (3.09–3.37)	4,644.75	3.94	3.96 (3.85–4.07)	0.66 (0.65–0.68)
Central Sub-Saharan Africa	819.98	3.61	4.69 (4.37–5.03)	2,448.76	4.07	5.01 (4.81–5.21)	0.21 (0.18–0.23)
East Asia	57,719.16	9.09	10.34 (10.25–10.42)	82,646.78	14.39	12.27 (12.19–12.36)	0.56 (0.54–0.57)
Eastern Europe	5,011.64	5.03	4.76 (4.63–4.89)	5,413.39	6.62	5.27 (5.13–5.42)	0.32 (0.31–0.34)
Eastern Sub-Saharan Africa	2,739.11	3.52	4.66 (4.48–4.84)	7,735.88	3.98	5.02 (4.9–5.13)	0.24 (0.23–0.25)
High-income Asia Pacific	7,079.48	8.72	8.21 (8.02–8.4)	7,166.14	11.26	9.19 (8.97–9.41)	0.36 (0.35–0.37)
High-income North America	20,229.54	15.16	14.14 (13.95–14.34)	37,137.68	25.33	23.5 (23.27–23.75)	1.65 (1.63–1.67)
North Africa and Middle East	6,403.54	4.31	5.36 (5.22–5.49)	19,771.59	6.63	6.39 (6.3–6.48)	0.56 (0.55–0.58)
Oceania	215.63	7.3	8.91 (7.75–10.2)	550.31	8.58	9.39 (8.62–10.21)	0.17 (0.12–0.21)
South Asia	19,343.04	3.99	4.66 (4.6–4.73)	42,662.76	4.7	4.97 (4.93–5.02)	0.22 (0.21–0.22)
Southeast Asia	14,043.92	6.42	7.72 (7.59–7.85)	31,287.55	9.59	9.29 (9.18–9.39)	0.61 (0.6–0.62)
Southern Latin America	1,928.71	8.78	9.26 (8.85–9.68)	3,643.09	11.98	11.48 (11.11–11.85)	0.67 (0.65–0.7)
Southern Sub-Saharan Africa	1,095.86	4.58	5.74 (5.4–6.1)	2,418.78	6.19	6.3 (6.05–6.56)	0.3 (0.29–0.32)
Tropical Latin America	2,251.16	3.12	3.54 (3.39–3.69)	5,081.27	4.83	4.48 (4.35–4.6)	0.75 (0.74–0.77)
Western Europe	11,127.84	6.52	6.25 (6.13–6.36)	12,799.75	8.06	6.98 (6.86–7.1)	0.35 (0.34–0.36)
Western Sub-Saharan Africa	3,092.88	3.91	4.95 (4.78–5.13)	8,835.26	4.16	5.24 (5.13–5.35)	0.2 (0.19–0.21)

ASDR, age-standardized DALYs rate; CI, confidence interval; AAPC, average annual percentage change.

### Gout burden in young people by SDI region

In 2021, the lowest ASPR, ASIR, and ASDR were observed in the low SDI region, at 147.02 (95% CI: 146.64–147.39) per 100,000 persons, 36.96 (95% CI: 36.78–37.15) per 100,000 persons, and 4.92 (95% CI: 4.85–4.99) per 100,000 person-years, respectively. The highest ASPR, ASIR, and ASDR were observed in the high SDI region, at 406.07 (95% CI: 405.51–406.63) per 100,000 persons, 83.55 (95% CI: 83.29–83.81) per 100,000 persons, and 13.36 (95% CI: 13.26–13.47) per 100,000 person-years, respectively. From 1990 to 2021, ASPR, ASIR, and ASDR increased across all SDI regions, indicating that the burden of gout in young people increased to varying degrees across all SDI regions. Notably, the largest increases in ASPR and ASIR were observed in the high SDI region at 40.8%, 39.7%, and 34.9%, respectively. In comparison, the smallest increases were observed in the low SDI region at 6.1%, 6.3%, and 6.7%, respectively (Tables 1–3).

### Gout burden in young people across 21 GBD regions

**Fig 4:**
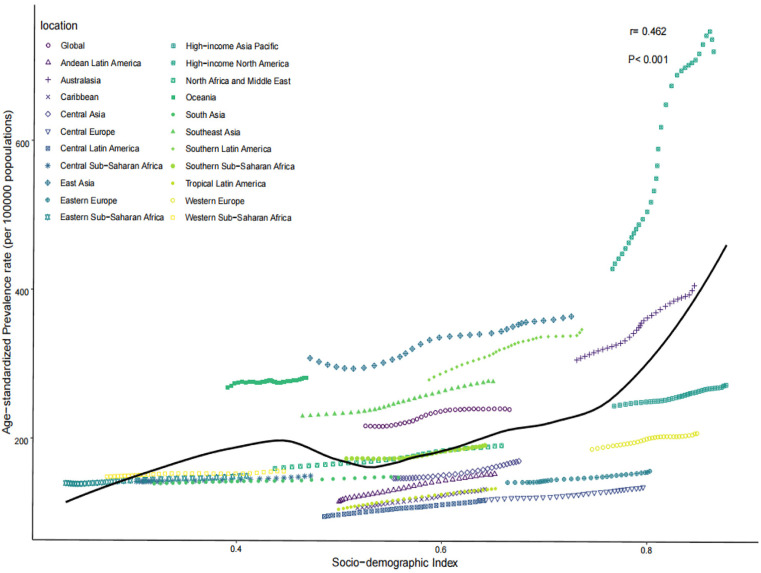
ASPR of the 21 GBD regions in 2021. ASPR, age-standardized prevalence rate; GBD, global burden of disease.

**Fig 5:**
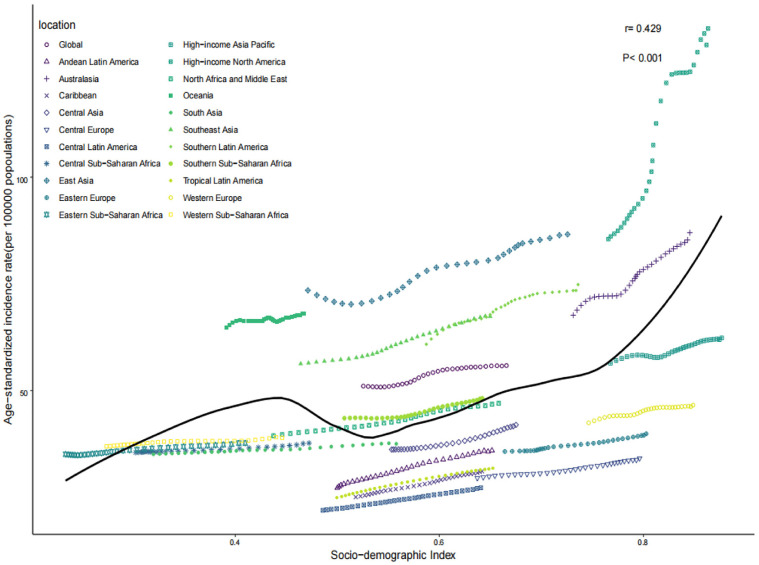
ASIR of the 21 GBD regions in 2021. ASIR, age-standardized incidence rate; GBD, global burden of disease.

**Fig 6:**
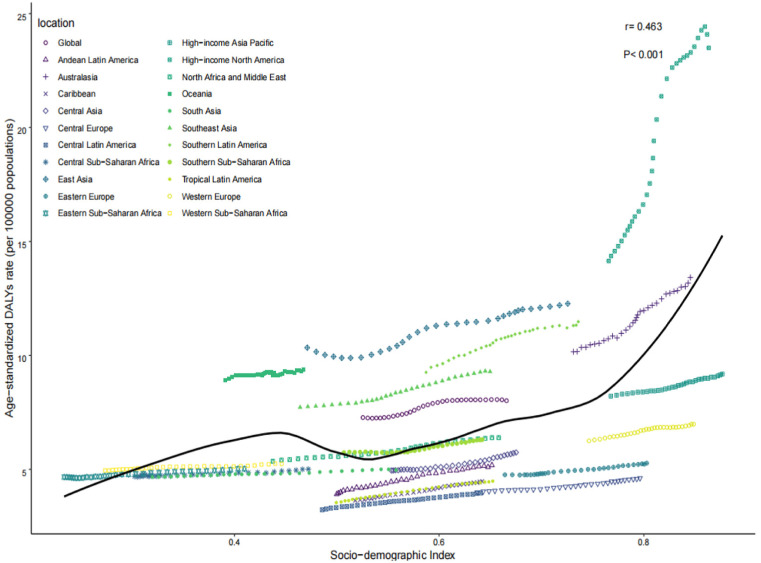
ASDR of the 21 GBD regions in 2021. ASDR, age-standardized DALYs rate; GBD, global burden of disease.

In 2021, ASPR, ASIR, and ASDR among the 21 GBD regions positively correlated with SDI (*r* = 0.462, 0.429, 0.463, *P* < 0.001). The highest ASPR, ASIR, and ASDR were observed in the high-income South America region, at 722.2 (95% CI: 720.93–723.58) per 100,000 persons, 134.81 (95% CI: 134.23–135.38) per 100,000 persons, and 23.5 (95% CI: 23.27–23.75) per 100,000 person-years, respectively. The second highest was observed in the Australasia region, at 407.64 (95% CI: 404.27–411.03) per 100,000 persons, 86.99 (95% CI: 85.43–88.58) per 100,000 persons, and 13.42 (95% CI: 12.82–14.05) per 100,000 person-years, respectively. The lowest ASPR, ASIR, and ASDR were observed in the Central Latin America region, at 117.98 (95% CI: 117.36–118.61) per 100,000 persons, 27.23 (95% CI: 26.94–27.53) per 100,000 persons, and 3.96 (95% CI: 3.85–4.07) per 100,000 person-years, respectively (Tables 1–3 and Figs 4–6).

### Global burden of gout among young adults, stratified by sex

In 2021, ASPR, ASIR, and ASDR for gout in young men worldwide were 386.67 (95% CI: 386.38–386.96) per 100,000 persons, 136.04 (95% CI: 135.64–136.44) per 100,000 persons, and 12.86 (95% CI: 12.81–12.92) per 100,000 person-years, respectively. For young women, these rates were 91.36 (95% CI: 91.22–91.51) per 100,000 persons, 32.7 (95% CI: 32.49–32.90) per 100,000 persons, and 3.06 (95% CI: 3.04–3.09) per 100,000 person-years, respectively, with males having significantly higher rates than females. From 1990 to 2021, the increases in ASPR, ASIR, and ASDR for young men were 19.4%, 18.5%, and 11.5%, respectively, whereas those for young women were 17.2%, 16.5%, and 6.6%, respectively. with a notably higher increase in ASDR for males (Tables 1–3).

### Gout-related DALYs attributable risk factors

**Fig 7:**
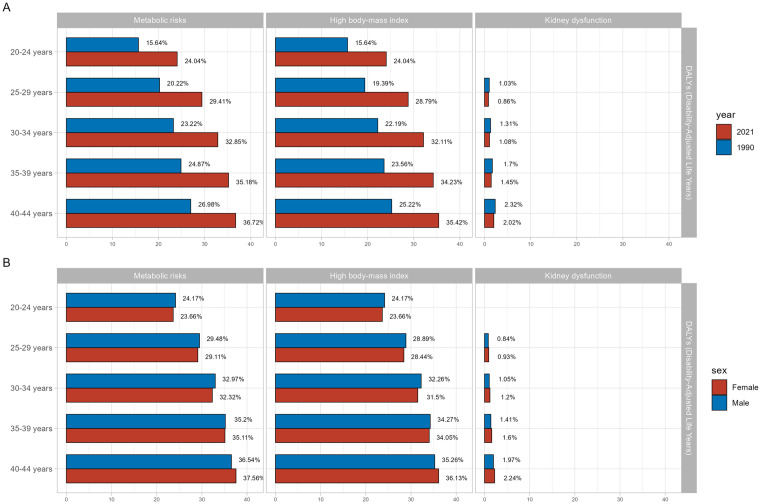
Risk factors contributing to gout-related DALYs globally and across age groups. (A) Risk factors in 2021 compared to 1990 that contributed to gout-related DALYs worldwide. (B) Risk factors in females compared to males that contributed to gout-related DALYs globally. DALYs, disability-adjusted life years.

A search of the GBD database found that the leading contributors to gout-related DALYs were metabolic risk factors, including high body mass index (BMI) and kidney dysfunction. However, BMI risk factors were not available for individuals aged 15–20 years, and data on kidney dysfunction were absent for those aged 15–25 years. Therefore, 20–44 and 25–44 years were selected for the visual analysis of BMI and kidney dysfunction as risk factors, respectively (Fig 7).

### Decomposition analysis of gout burden

**Fig 8:**
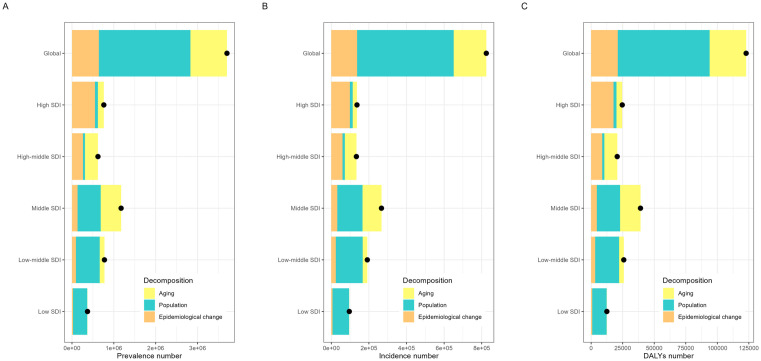
Decomposition analysis of gout indicators from 1990 to 2021. (A) Prevalence number, (B) Incidence number, (C) DALY number. Black dots: overall change values of population growth, aging, and epidemiological change.

In 1990 and 2021, young people worldwide aged 25–44 years had a significantly higher DALY rate for gout caused by high BMI than that caused by kidney dysfunction. However, the DALY rate for gout caused by high BMI was significantly higher in 2021 than in 1990. For individuals aged 25–44 years, the DALY rate for gout caused by kidney dysfunction was lower in 2021 than in 1990. Notably, the DALY rate for gout caused by high BMI was comparable between men and women, whereas that caused by kidney dysfunction was slightly higher in women than in men (Tables 4–6, Fig 8).

**Table 4:** Changes in prevalence number according to population-level determinants and causes from 1990 to 2021.

Socio-demographic Index	Overall difference	Aging	Population	Epidemiological change
Global	3,702,747.00	875,258.80 (23.64%)	2,183,904.68 (58.98%)	643,583.52 (17.38%)
SDI				
High SDI	760,594.01	139,938.78 (18.40%)	72,320.68 (9.51%)	548,334.56 (72.09%)
High-middle SDI	620,762.54	309,032.56 (49.78%)	48,313.73 (7.78%)	263,416.25 (42.43%)
Middle SDI	1,173,345.87	489,376.93 (41.71%)	550,255.24 (46.90%)	133,713.70 (11.40%)
Low-middle SDI	775,375.96	111,779.72 (14.42%)	566,755.27 (73.09%)	96,840.96 (12.49%)
Low SDI	370,590.81	6,111.00 (1.65%)	340,646.86 (91.92%)	23,832.96 (6.43%)

**Table 5:** Changes in incidence numbers according to population-level determinants and causes from 1990 to 2021.

Socio-demographic Index	Overall difference	Aging	Population	Epidemiological change
Global	824,329.15	173,228.12 (21.01%)	513,649.86 (62.31%)	137,451.17 (16.67%)
SDI				
High SDI	136,537.10	23,066.88 (16.89%)	14,896.00 (10.91%)	98,574.23 (72.20%)
High-middle SDI	133,375.54	61,931.44 (46.43%)	11,445.72 (8.58%)	59,998.38 (44.98%)
Middle SDI	266,739.27	101,199.98 (37.94%)	133,752.18 (50.14%)	31,787.11 (11.92%)
Low-middle SDI	191,581.86	24,425.28 (12.75%)	143,089.69 (74.69%)	24,066.70 (12.56%)
Low SDI	95,613.48	1,235.10 (1.29%)	88,011.13 (92.05%)	6,367.25 (6.66%)

**Table 6:** Changes in DALY number according to population-level determinants and causes from 1990 to 2021.

Socio-demographic Index	Overall difference	Aging	Population	Epidemiological change
Global	122,841.44	28,896.99 (23.52%)	72,866.17 (59.32%)	21,078.29 (17.16%)
SDI				
High SDI	24,661.98	4,569.03 (18.53%)	2,385.54 (9.67%)	17,707.41 (71.80%)
High-middle SDI	20,666.95	10,233.77 (49.52%)	1,616.77 (7.82%)	8,816.42 (42.66%)
Middle SDI	39,114.54	16,236.71 (41.51%)	18,422.75 (47.10%)	4,455.08 (11.39%)
Low-middle SDI	25,872.84	3,702.22 (14.31%)	18,998.03 (73.43%)	3,172.60 (12.26%)
Low SDI	12,455.92	199.56 (1.60%)	11,394.67 (91.48%)	861.70 (6.92%)

DALYs, disability-adjusted life years.

### Forecast analysis for the global burden of gout among young people over the next 25 years

**Fig 9:**
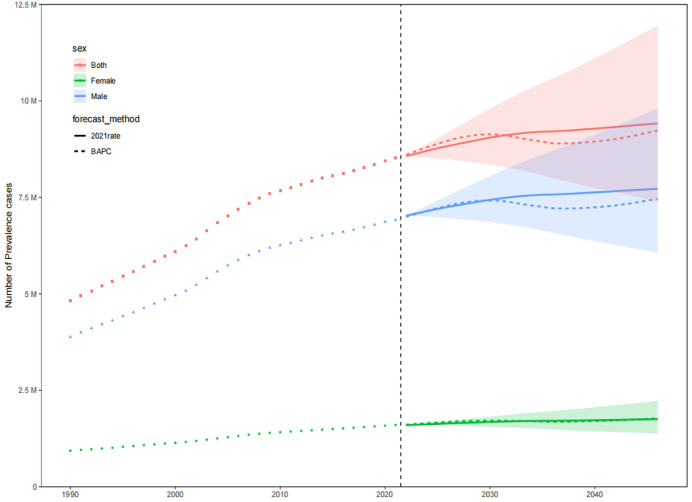
Prediction of the prevalence number over the next 25 years.

**Fig 10:**
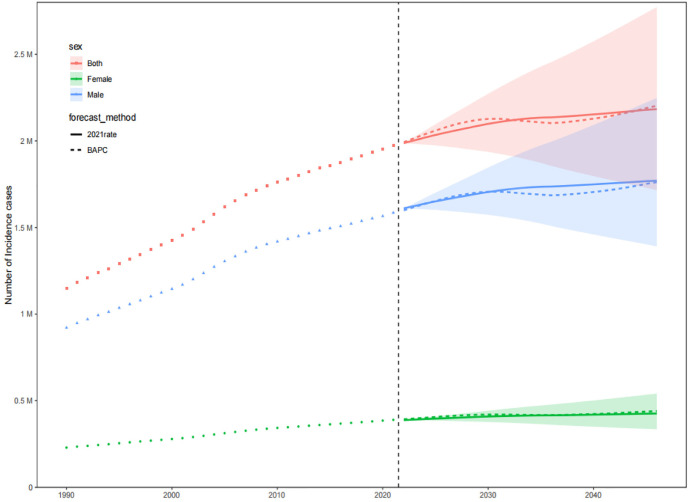
Prediction of the incidence number over the next 25 years.

**Fig 11:**
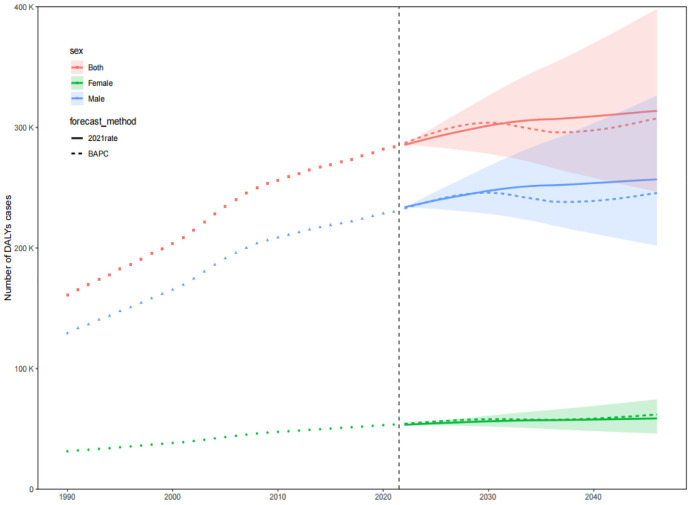
Prediction of the number of DALYs over the next 25 years.

**Fig 12:**
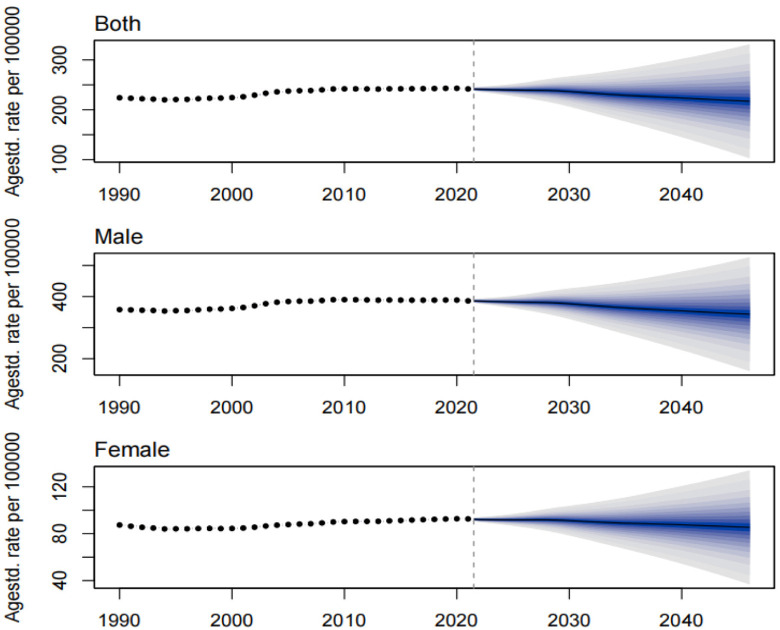
Prediction of the prevalence rate over the next 25 years.

**Fig 13:**
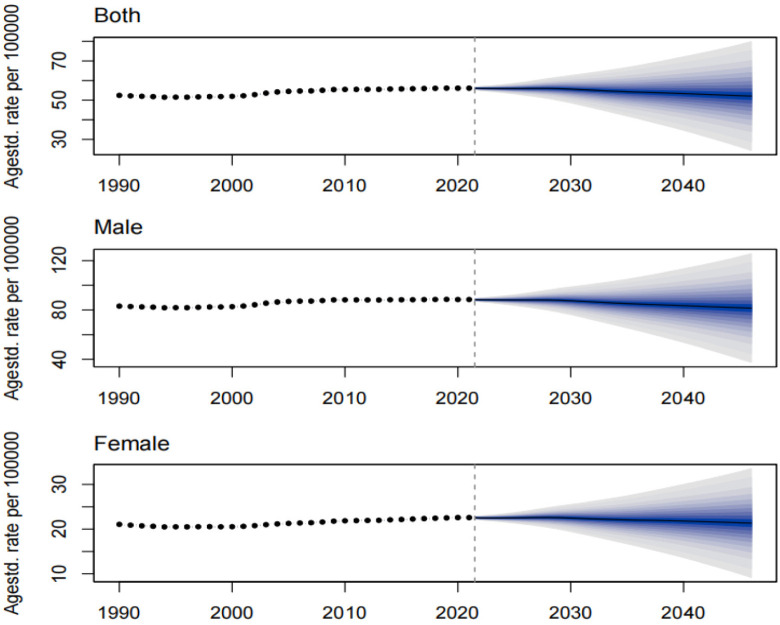
Prediction of the incidence rate over the next 25 years.

**Fig 14:**
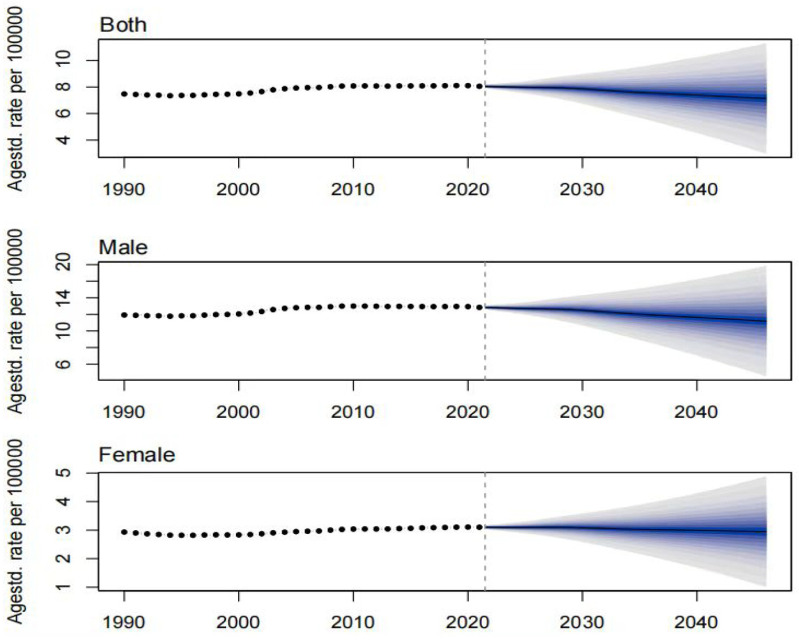
Prediction of the DALY rate over the next 25 years.

The prevalence, incidence, and DALYs for gout in young people are expected to vary over time and across regions. By 2046, the global prevalence, incidence, and DALYs for gout in young people are projected to reach 217.15 per 100,000 persons, 52.08 per 100,000 persons, and 7.14 per 10 person-years, respectively. For males, these figures will be 343.46 per 100,000 persons, 81.53 per 100,000 persons, and 11.20 per 100,000 person-years, respectively, and for females, 85.40 per 100,000 persons, 21.35 per 100,000 persons, and 2.95 per 100,000 person-years, respectively. The projected number of individuals with gout, incident cases, and DALYs for young people globally will be approximately 9,229,870, 2,202,802, and 307,441 person-years, respectively. Among these, males will account for 7,460,349 individuals, 1,762,412 incident cases, and 245,542 person-years, while females will account for 1,769,521 individuals, 440,390 incident cases, and 61,899 person-years. Over the next 25 years, the global number of young people with gout, incident cases, and DALYs is expected to continue rising, primarily driven by an increase in the number of cases among males (Figs 9–14).

## Discussion

This study analyzed the burden of gout among young people across 204 countries and territories and 21 regions from 1990 to 2021 using data from the GBD database and projected trends for the next 25 years. The results indicate that the ASPR, ASIR, and ASDR for gout in young people globally were significantly higher in 2021 compared to 1990. The burden of gout is expected to continue growing over the next 25 years. Consequently, the burden of gout among young people has been rising, becoming a significant global public health issue.

The analysis identified clear sex differences in young people with gout, with lower incidence, prevalence, and DALYs in females. This disparity may be related to the regulatory role of estrogen on urate transporter expression, leading to increased uric acid excretion and reduced risk of gout [15,16]. Dietary patterns also influence the risk of developing gout. Males with gout are more likely to have diets high in seafood, beef, pork, and alcohol [17]. A prospective study of 730 male patients found an association between alcohol consumption and increased risk of gout [18]. However, for young people, the primary factor contributing to sex-based differences is likely estrogen in women of reproductive age. While the incidence, prevalence, and DALYs among young females remain lower than those of males, they have also increased since 1990. Symptomatically, women are more likely to experience upper limb involvement, which may cause delayed diagnosis and treatment [19]. Furthermore, risk factors, such as age, medication use, and comorbidities, differ between men and women, resulting in variations in clinical characteristics [20]. The current study showed that the DALY rate for gout related to kidney dysfunction was slightly higher in women than in men. Women who developed gout at older ages are at increased risk for endocrine, metabolic, cardiovascular, and kidney comorbidities [21]. Additionally, mortality risk associated with gout is reportedly higher in women than in men [22,23]. These findings indicate that research focused on prevention and management of gout in women may be warranted, as previous studies have predominantly concentrated on male populations.

Gout is a metabolic disease that exhibits a strong association with kidney dysfunction and high BMI. This study demonstrated that the DALY rate for gout caused by high BMI significantly exceeded that caused by kidney dysfunction in 1990 and 2021, with the impact of high BMI becoming more pronounced in 2021. Moreover, the effect of high BMI as a risk factor for gout was relatively consistent among young males and females. Notably, individuals with obesity (BMI > 30 kg/m^2^) face more than twice the risk of developing gout compared to those with a BMI below 30 kg/m^2^ [1,24]. A prospective study of 44,654 men without a prior history of gout demonstrated that a combination of hypertensive dietary methods, abstaining from alcohol, and avoiding diuretics reduced the incidence of gout by over 50% among men who were overweight or of normal weight. However, this preventive approach did not significantly reduce new gout episodes among those with obesity, suggesting that obesity independently elevates the risk of gout beyond the benefits conferred by positive dietary patterns [25].

Recent years have witnessed a marked increase in gout prevalence across East Asian countries, driven by rapid nutritional transitions accompanying rapid economic development [26–28]. For example, consumption of ultra-processed foods, which is positively correlated with obesity [29], accounts for approximately 45% of the daily caloric intake among Canadian adults [30]. The adoption of a westernized lifestyle—characterized by high-purine diets and sedentary behavior—has contributed to rising obesity rates among younger populations. Moreover, young people with genetic predispositions are at an increased risk of developing gout at an early age. In developed countries, most DALYs are now attributable to loss of functional health rather than premature mortality [31]. Addressing obesity can reduce the incidence of gout in men [32]. As such, public health initiatives should prioritize education on maintaining healthy uric acid levels through lifestyle interventions, including weight management, regular physical exercise, and dietary modification [33].

This study demonstrates that higher SDI levels are associated with increased prevalence, incidence, and DALYs of gout among young people, with economically developed countries, such as the United States and Canada, experiencing a disproportionate burden. This suggests that socioeconomic and demographic factors significantly impact gout-related health outcomes in young people. The high presence of gout risk factors—such as high BMI—in these regions may partially account for the observed increase in age-standardized DALYs associated with gout in high SDI areas. Nevertheless, substantial variability in socioeconomic resources and gout risk remains even among high SDI nations. For instance, socioeconomic disparities, such as poverty, largely explain differences in gout prevalence between black and white adults in the United States [34]. Furthermore, there is notable geographical variation in gout incidence, which corelates with local dietary habits. For example, higher consumption of meat and certain seafood in Europe and the United States contributes to greater gout incidence compared to Asia, where traditional diets are based on rice and vegetables [35,36]. However, in recent years, the burden of gout has increased rapidly in several East Asian countries undergoing significant socioeconomic development and shifts toward Western-style diets, including increased intake of fructose-sweetened beverages, alcohol, and purine-rich foods [37–39]. In East Asia, the number of individuals with gout has increased by 1.76 times over the past 30 years [37]. Notably, a recent study in China reported a doubling in gout prevalence among young people within the past ten years, strongly correlated with urbanization and rising obesity trends [38]. Similar trends have been reported in Japan, where traditional diets are increasingly replaced by processed foods and sugary drinks [39]. Collectively, these observations emphasize the critical influence of modifiable environmental factors on the global gout epidemic.

Decomposition analysis further revealed that population growth predominantly drives increases in gout-related indicators, while changes in gout burden differ according to developmental and geographical contexts. Consequently, it is essential for regions at varying developmental stages within the same country to adopt context-specific strategies to mitigate the burden of gout.

Prediction analysis for the next 25 years suggests that the global burden of gout among young people is unlikely to decline, particularly among young men. Gout can cause severe pain, functional impairment, and reduced workplace participation. Compared to those without gout, individuals with gout are more likely to have increased work absences [40], which can result in financial and social effects for patients and their families or communities. The comorbidities commonly associated with gout, such as hypertension, diabetes, cardiovascular disease, and kidney disease, further exacerbate the overall disease burden, contributing to higher incidence and mortality rates [41,42]. This remains a significant challenge for healthcare systems. Therefore, additional measures may be necessary to address the prevalence of gout among young people.

This study had several limitations. Although the GBD model accounts for different case definitions, some bias in the estimates of gout burden may remain due to the use of data sources with variable quality. First, the clinical manifestations of gout can overlap with those of acute calcium pyrophosphate crystalline arthritis (called pseudogout), potentially leading to overestimation of gout prevalence. A definite diagnosis requires further examination of joint fluid and synovial fluid, yet access to such resources may be limited in low-income areas, increasing the potential for statistical bias in diagnosing gout among young populations. Second, misclassification is possible, particularly at disease onset when gout may be mistaken for other conditions, such as sprains or infections, leading to delayed or incorrect diagnoses. Additionally, other forms of inflammatory arthritis may be misdiagnosed as gout. Third, updates to the diagnostic criteria for gout have occurred; the original 1977 ACR classification has been replaced by the 2015 ACR/European League Against Rheumatism classification [12]. Consequently, GBD estimates of gout among young people may differ from actual data. Future research should consider these methodological differences to enhance the accuracy and comparability of gout burden assessments across regions. Despite these limitations, the 2024 update of the GBD database, along with recent epidemiological data, is valuable for health system departments to formulate effective prevention and control strategies and address related risks.

This study provides a comprehensive assessment of the growing global burden of gout in young people. The observed increase in cases from 1990 to 2021 challenges the traditional perception of gout as an “older adult disease,” highlighting unmet prevention needs earlier in life. Projections for 2050 suggest that gout could become a leading cause of early-life musculoskeletal disability, potentially accompanied by metabolic and cardiovascular comorbidities. Future research should address factors contributing to the progression of hyperuricemia to symptomatic gout earlier in certain populations and evaluate age-stratified interventions targeting high-risk young people using dietary modulation or early urate-lowering therapy. The development of cost-effective primary care screening models for early detection is also required. Key proposed studies include: prospective cohort designs (e.g., birth cohort with regular uric acid type tests) to optimize intervention timing; integration of genome-wide association studies with dietary and socioeconomic data targeting high-incidence populations; and randomized controlled trials assessing game-based lifestyle interventions or wearable-assisted uric acid monitoring in young people with preclinical hyperuricemia. The Epidemiological data presented in this study can help support public health authorities in developing targeted strategies to mitigate the occurrence of gout.

## Abbreviations

AAPC: average annual percentage change
ASDR: age-standardized DALYs rate
ASIR: age-standardized incidence rate
ASPR: age-standardized prevalence rate
BAPC: Bayesian age-period-cohort
CI: confidence interval
DALYs: disability-adjusted life years
GBD: global burden of disease
SDI: socio-demographic index
BMI: body mass index
